# The Fecal Bacterial Microbiota in Horses with Equine Recurrent Uveitis

**DOI:** 10.3390/ani11030745

**Published:** 2021-03-09

**Authors:** Michelle Martin de Bustamante, Diego Gomez, Jennifer MacNicol, Ralph Hamor, Caryn Plummer

**Affiliations:** 1 Department of Small Animal Clinical Sciences, College of Veterinary Medicine, University of Florida, Gainesville, FL 32610, USA; michellemartinde@ufl.edu (M.M.d.B.); hamor@ufl.edu (R.H.); 2 Department of Large Animal Clinical Sciences, College of Veterinary Medicine, University of Florida, Gainesville, FL 32610, USA; dgomezni@uoguelph.ca; 3 Department of Clinical Studies, Ontario Veterinary College, University of Guelph, Guelph, ON N1G 2W1, Canada; 4 Department of Animal Biosciences, Ontario Agriculture College, University of Guelph, Guelph, ON N1G 2W1, Canada; jmacnico@uoguelph.ca

**Keywords:** anterior uveitis, ERU, fecal microbiota, high-throughput sequencing, horse, microbiome, metabolome

## Abstract

**Simple Summary:**

Equine recurrent uveitis (ERU) is an immune-mediated disease characterized by recurrent episodes of intraocular inflammation. Despite being a major cause of blindness in horses worldwide, the exact pathogenesis of ERU remains unknown. Recently, changes in the normal balance of the gastrointestinal tract bacteria (also known as dysbiosis) have been described in several immune-mediated diseases in humans, including uveitis. As such, the objective of this study was to compare the fecal bacterial community (the fecal microbiota) of horses with ERU with that of healthy horses living on the same farms. Study results revealed no significant alterations in the fecal microbiota between horses with ERU and healthy horses housed in the same environment. In order to better determine if there is an association between dysbiosis and ERU, future investigations are warranted to more specifically compare the fecal microbiota of horses that are having active flare-ups of ERU with that of horses in a quiescent period of the disease and with healthy horses.

**Abstract:**

The objective of this study was to describe and compare the fecal bacterial microbiota of horses with equine recurrent uveitis (ERU) and healthy horses using next-generation sequencing techniques. Fecal samples were collected from 15 client-owned horses previously diagnosed with ERU on complete ophthalmic examination. For each fecal sample obtained from a horse with ERU, a sample was collected from an environmentally matched healthy control with no evidence of ocular disease. The Illumina MiSeq sequencer was used for high-throughput sequencing of the V4 region of the 16S rRNA gene. The relative abundance of predominant taxa, and alpha and beta diversity indices were calculated and compared between groups. The phyla Firmicutes, Bacteroidetes, Verrucomicrobia, and Proteobacteria predominated in both ERU and control horses, accounting for greater than 60% of sequences. Based on linear discriminant analysis effect size (LEfSe), no taxa were found to be enriched in either group. No significant differences were observed in alpha and beta diversity indices between groups (*p* > 0.05 for all tests). Equine recurrent uveitis is not associated with alteration of the gastrointestinal bacterial microbiota when compared with healthy controls.

## 1. Introduction

Equine recurrent uveitis (ERU) is an immune-mediated disease characterized by spontaneously recurrent episodes of intraocular inflammation, with an estimated prevalence of 1 to 2% in the United States [1]. Horses suffering acute flare-ups experience blepharospasm, epiphora, photophobia, and ocular hypotension. With chronicity, ERU can lead to the development of cataracts, secondary glaucoma, retinal detachment, or phthisis bulbi secondary to chronic inflammation. ERU is a major cause of ocular pain, blindness, and globe loss in horses worldwide [1,2,3,4]. Consequently, it can have a significant impact on horse welfare, owner wellbeing, and the productivity of the equine industry due to decreased performance, financial loss, change of ownership, and even euthanasia [1]. Treatment is symptomatic, primarily aimed at reducing inflammation, improving comfort, and preventing future flare-ups. Though ERU is an immune-mediated, T cell-driven disease, the exact pathogenesis remains unknown [2,5].

Equine recurrent uveitis shares many characteristics with a disease that occurs in humans, autoimmune uveitis, and serves as the only known naturally occurring, spontaneous animal model for the disease [2,6,7]. In human and horses, there is limited knowledge of the etiology and pathogenesis of autoimmune uveitis. As such, therapeutic options are limited. In humans and animals, alteration of the gut microbiota (also known as dysbiosis) is linked with several immune-mediated diseases, including rheumatoid arthritis, type I diabetes, colitis, celiac disease, and inflammatory bowel disease [8,9,10,11,12,13,14]. Similarly, the gut microbiota appears to play a role in autoimmune uveitis in humans [14,15,16,17]. Differences in the abundance of certain gut bacterial communities (at the genus level) were identified between healthy humans and those with autoimmune uveitis [16,17], and modulation of the gut bacterial microbiota by using broad-spectrum oral antibiotics decreased severity of autoimmune uveitis in a mouse model [14]. Based on this information, some authors hypothesize that gastrointestinal microbiota can play an important role in development of autoimmune uveitis. 

The fecal bacterial microbiota of normal horses has been previously characterized using next-generation sequencing technology [18,19,20,21], but studies investigating the gut bacteria microbiota in horses with autoimmune diseases, in particular ERU, are lacking. This study aims to investigate the fecal bacterial microbiota of horses with ERU and to compare it with that of environment-matched healthy horses. We hypothesized that the fecal bacterial microbiota composition would be significantly different in horses with ERU compared with healthy control horses, with specific taxa being enriched in ERU horses.

## 2. Materials and Methods

### 2.1. Ethical Considerations

This study was performed in accordance with the guidelines of the Institutional Animal Care and Use Committee of the University of Florida (IACUC Study #201810412). Written owner consent was obtained for study enrollment.

### 2.2. Sample Size Calculation

The sample size was determined using previously published guidelines on biostatistical methods for analysis of microbiota data (Dirichlet multinomial distribution model) [22]. With an expected number of 10,000 to 20,000 sequence reads (per horse) available for comparison and an alpha of 5%, 15 subjects were required for a power of 80%. This sample size was also supported by the results from previous studies in which sample sizes of 15 animals (with an equal number of age-matched controls) were sufficient to yield significant differences in the relative abundance of the bacterial microbiota at all taxonomic levels (from phyla to genus level), and diversity indices [23,24,25].

### 2.3. Animals

Fecal samples were collected from 15 adult, greater than 1-year-old, client-owned horses, diagnosed with ERU based upon complete ophthalmic examination by a board-certified veterinary ophthalmologist and ophthalmology resident at the University of Florida Large Animal Hospital (UF-LAH). Horses were eligible for enrollment in the study in either clinically active or quiescent disease state. All ERU horses had a minimum of two previously documented episodes of acute anterior uveitis with no identifiable primary cause. Horses presenting with active uveitis at the time of sample collection had clinical signs consistent with an acute flare-up, such as aqueous flare, blepharospasm, epiphora, miosis, ocular hypotension, or vitreal cellular infiltrate. Horses presenting in a quiescent disease state had evidence of chronic uveitis on examination, including blunted corpora nigra, cataract, chorioretinal scarring, iris hyperpigmentation, lens subluxation or luxation, phthisis bulbi, pigment deposition on the anterior lens capsule, or posterior synechiae. For each fecal sample collected from a horse with ERU, a fecal sample was collected from a healthy control horse housed in similar environmental conditions on the same farm. Cases and controls were fed the same hay and had access to similar pastures, but the amount and type of grain supplemented differed in some pairs. Horses with a history of concurrent health problems or systemic antimicrobial therapy within the 6 months before fecal sample collection were excluded from the study.

### 2.4. Sample Collection and Processing

Fecal samples were collected at the time of each horse’s hospital visit. Once a bowel movement occurred, 2 to 3 g of feces was collected from the middle of a fecal pile. The samples were immediately packaged, labeled, and stored in a −80 °C freezer until processing. Once thawed, bacterial DNA was extracted from the samples using a commercially available kit (EZNA Stool DNA Kit). DNA extraction was performed as previously described [25]. Following extraction, DNA was amplified with a set of oligonucleotide primers targeting the V4 region of the 16S rRNA gene with overhanging adapters for annealing to Illumina universal index sequencing adaptors [26]. The library pool was sequenced with an Illumina MiSeq (Illumina RTA v1.17.28; MCS v2.2) for 250 cycles from each end.

### 2.5. Data Analysis

Bioinformatic analysis was completed using Mothur software (https://mothur.org, accessed on 20 December 2020) [27] with a previously published protocol [28,29]. Sequences underwent quality control filtering, identification using the Ribosomal Database Project classifier, and were binned into phylotypes [30,31]. Random subsampling was completed to normalize the sequence count. Good’s coverage index was used to assess sampling coverage. Changes in fecal microbiota were evaluated using the health status (ERU and healthy) as the main exposure of interest. Alpha (α-) diversity was examined using the inverse Simpson’s (diversity), Shannon’s evenness (evenness), and Chao-1 (richness) indices. Data for Chao-1 and inverse Simpson’s indices were non-normally distributed, and therefore log transformation was performed. Comparison between groups was performed using a *t*-test. Community membership and structure were measured with the Jaccard and the Yue and Clayton indices, respectively, and compared statistically with an analysis of molecular variance test (AMOVA) and a parsimony test [23,24,25]. Dendrograms and PCoA plots were developed based on the Yue and Clayton and the Jaccard indices. Relative abundances of the main phyla, classes, orders, families, and genera were calculated. Linear discriminant analysis effect size (LEfSe) determined the bacterial taxa enriched in feces of each group, based on *p* < 0.05 and a linear discriminant analysis (LDA) score >3.0 [32]. Dirichlet multinomial mixture model (DMM) was used to assess the number of different meta-communities into which the data could be clustered [33].

## 3. Results

### 3.1. Animals

The study population of 15 horses with ERU included 7 geldings, 7 mares, and 1 stallion, all ranging in age from 6 to 28 years (median 16 years). The following breeds were represented in the ERU group: Appaloosa (*n* = 6), Quarter Horse (*n* = 4), American Paint Horse (*n* = 1), Draft (*n* = 1), Dutch Warmblood (*n* = 1), Miniature Horse (*n* = 1), and Thoroughbred (*n* = 1). The environmentally matched, healthy controls included 6 mares, 4 geldings, and 5 horses of unspecified sex. The control horses ranged in age from 4 to 23 years (median 14.5 years). The following breeds were represented in the control group: Appaloosa (*n* = 3), American Paint Horse (*n* = 2), Warmblood (*n* = 2), Arabian (*n* = 1), Quarter Horse (*n* = 1), Thoroughbred (*n* = 1), Connemara Pony (*n* = 1), and mixed breeds (*n* = 4). Horses with ERU were treated with a variety of ophthalmic and systemic medications based upon the severity of clinical disease and clinician preference. Patient signalment, affected eye(s), pertinent ophthalmic examination findings, and treatment at the time of sample collection are detailed in Table 1.

### 3.2. Sequence Analysis

The total number of raw sequences was 6,193,460. A total of 4,321,050 good-quality sequences were used for the final analysis (mean sequences per sample: 143,951 per sample; SD: 24,098; median: 138,970; range: 89,925 to 212,222). The sequences obtained from horses were rarified to an even sequencing depth of 89,900 sequences per sample to adjust for uneven sequencing depth across the samples.

### 3.3. Fecal Microbiota of Healthy and ERU Horses

#### 3.3.1. Alpha Diversity

**Figure 1:**
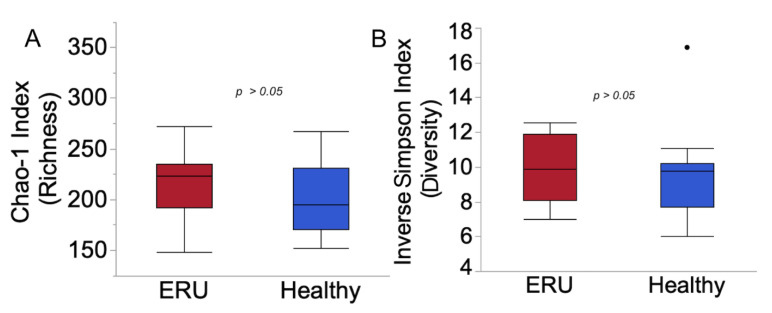
Alpha diversity measurements in horses with equine recurrent uveitis (*n* = 15) and environmentally matched healthy controls (*n* = 15). (**A**) Estimated diversity (inverse Simpson’s diversity index; (**B**) estimated richness (Chao-1 index).

The mean inverse Simpson’s (Figure 1A), Shannon’s evenness, and Chao-1 (Figure 1B) indices were similar between healthy horses and horses with ERU (*p* > 0.05, for all comparisons).

#### 3.3.2. Relative Abundance and LEfSe Analysis

**Figure 2:**
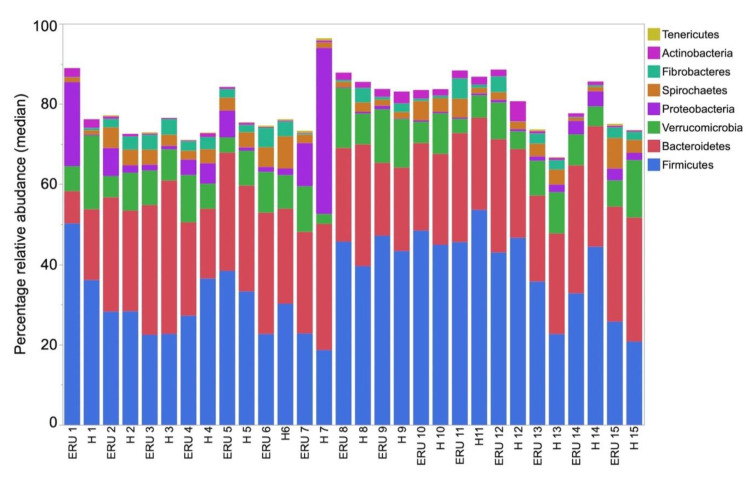
Median relative abundance of the main bacterial phyla in the feces of individual horses with equine recurrent uveitis (ERU) and environmentally matched healthy controls (H) (*n* = 15 per group).

**Figure 3:**
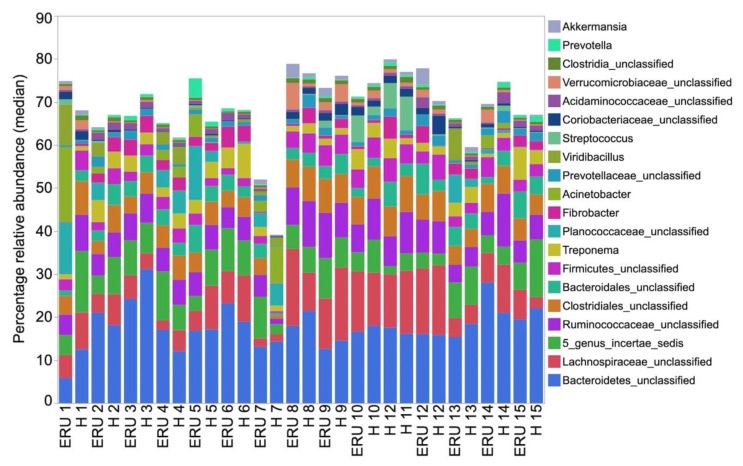
Median relative abundance of the main bacterial genera in the feces of individual horses with equine recurrent uveitis (ERU) and environmentally matched healthy controls (H) (*n* = 15 per group).

Twenty-two different phyla were identified, but Firmicutes, Bacteroidetes, Verrucomicrobia, and Proteobacteria accounted for more than 60% of total sequences (Figure 2). Firmicutes and Bacteroidetes each accounted for more than 20% of the total number of sequences. Forty-nine different classes, 96 orders, and 218 families were identified, but only 12, 13, and 16 accounted for ≥1% of sequences overall, respectively. In total, 592 genera were detected. Seventy-six and 15 of those were present at a relative abundance of >0.05% and >1%, respectively. The relative abundances of the most abundant phyla and genera found in healthy and ERU horses are presented in Figure 2 and Figure 3. LEfSe analysis failed to identify any taxa enriched in either healthy or ERU horses.

#### 3.3.3. Community Membership and Structure

**Figure 4:**
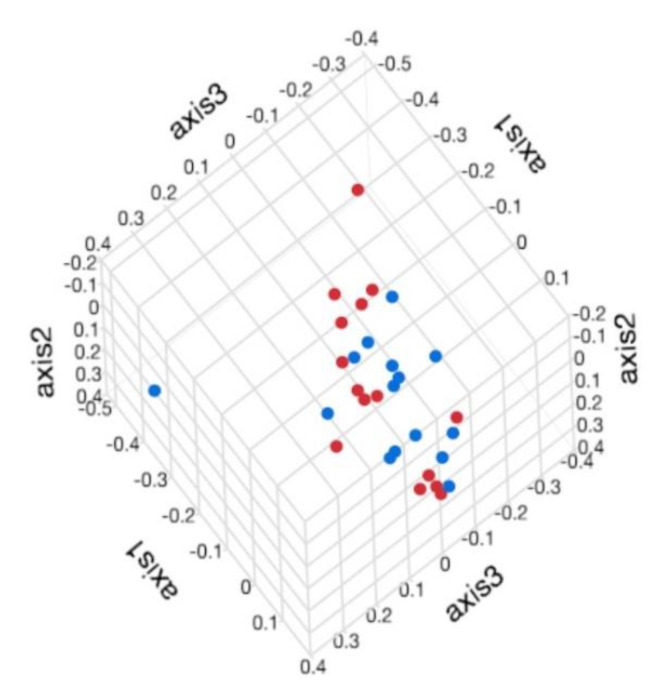
Three--dimensional principal coordinates analyses (PCoA) of the community membership (Jaccard index) of the fecal microbiota of horses with equine recurrent uveitis and environmentally matched healthy controls. Colored points indicate groups: healthy horses (red) and ERU horses (blue).

**Figure 5:**
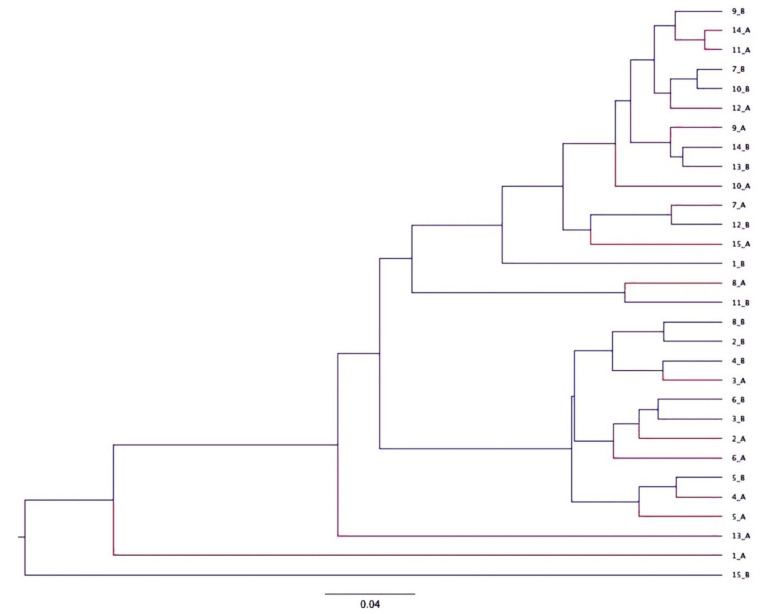
Dendrograms representing the similarity of community structure (Yue and Clayton index, right panel) found in fecal samples collected from horses with equine recurrent uveitis (red) and environmentally matched healthy controls (blue).

The community membership (Jaccard index) and structure (Yue and Clayton index) of healthy and ERU horses were similar (*p* > 0.05, for all comparisons). Distinct clusters (ERU versus healthy horses) were not visually evident on Jaccard and Yue and Clayton PCoA plots (Figure 4) or dendrograms (Figure 5), respectively.

#### 3.3.4. Meta-Community Analysis

Using the DMM, all samples were grouped into one community. Taxa differentiating samples from healthy and ERU horses were not identified.

## 4. Discussion

In the present study, no significant differences in the fecal bacterial microbiota were identified in the observed OTUs, and alpha and beta diversity between horses with ERU and healthy, environmentally matched controls. This stands in contrast to our hypothesized outcome, based upon the available literature, in which significant alterations in fecal microbiota were expected in horses with ERU compared with controls. Studies linking gastrointestinal dysbiosis to human autoimmune uveitis have centered on an inducible murine model of the disease, with limited data available to date evaluating the fecal microbiota of human patients with naturally acquired disease [14,15]. A small preliminary study documented that humans with chronic autoimmune uveitis, controlled with medication, have differences in the fecal microbiota composition at the genus level when compared with healthy controls [17]. A more recent study reported a higher microbial richness and diversity in healthy controls compared with humans with idiopathic uveitis and autoimmune uveitis [34]. Of interest, when the uveitis group was subdivided based upon underlying etiology (idiopathic versus autoimmune uveitis), the changes were similar in both groups. At the genus level, both idiopathic and autoimmune uveitis patients had differential enrichment of *Prevotella* and *Streptococcus* and a reduction in *Faecalibacterium*, *Bacteroides*, *Lachnospira*, and *Ruminococcus* when compared with healthy controls [34]. Bacteria of the genus *Faecalibacterium* are major producers of butyrate and play an important role in gastrointestinal (GI) physiology and health [35]. *Bacteroides*, *Lachnospira*, and *Ruminococcus* are also part of a healthy GI tract and serve a potential protective function; decreases in the abundance of bacteria belonging to the genus *Bacteroides* have been associated with autoimmune diseases including Crohn’s disease and rheumatoid arthritis [36]. The alteration in these important bacterial communities of the gastrointestinal tract of human patients with autoimmune uveitis led to the hypothesis that dysbiosis can play an important role in development of autoimmune uveitis. 

Dysbiosis is associated with dysregulation of the gut tight junction, which can result in bacteria, bacterial components (e.g., lipopolysaccharide), or bacterial byproducts translocating into systemic circulation. Those bacteria, bacterial components, or bacterial byproducts mimic retinal or ocular antigens, thereby predisposing the host to autoimmune uveitis [14,15,16,17]. Relatedly, some authors propose that specific gut bacteria may contribute to modulation of specific immune system responses (particularly related to T-regulatory cells), resulting in an increased susceptibility, overall, to immune-mediated disease processes [14,15,16,17]. Therefore, it is possible that maintenance of the normal balance of the gut microbiota would have beneficial effects (i.e., modulation of the immune system or recovery of tight junction integrity) in patients with autoimmune diseases. 

Similar to our study, one study investigating the gut microbiota composition in human patients with acute anterior uveitis (AAU) failed to identify significant differences in the fecal microbiota composition compared with healthy controls. However, the study did show that AAU patients had a unique fecal metabolic phenotype compared with controls [16]. Several fecal metabolites, including 6-deoxy-D glucose 1, linoleic acid, and palmitoleic acid, were increased in patients with AAU. Both gut microbiota and metabolome composition are important in immune homeostasis, and consequently may both play a role in the development of immune-mediated disease. The fecal bacterial microbiota can be similar between horses with ERU and healthy controls, but the fecal metabolome composition of horses with ERU could differ significantly from healthy controls. Future metabolomics studies are indicated to further investigate the fecal metabolic phenotype of ERU horses. 

Horses and humans have distinctly different gastrointestinal tract anatomy and physiology. Humans are monogastric omnivores, whereas horses are monogastric hindgut fermenting herbivores with a specialized large cecum and colon utilized for bacterial breakdown and utilization of fiber [37,38,39]. These anatomic and dietary differences are reflected in the unique fecal microbiota profiles found in the two species. In a study comparing the fecal microbiota of several different herbivorous and omnivorous mammals, the fecal microbiota of horses had significantly higher alpha diversity with a higher abundance of cellulolytic bacteria than humans. Additionally, on beta diversity analysis in the PCoA plot, horses and other herbivores clustered separately from humans [37]. These findings may account for the differences in results seen between the present study and the available literature characterizing the fecal microbiota in humans with autoimmune uveitis. Care should be taken when interpreting results from gut microbiota-related research and making comparisons between species, especially in those with distinct differences in gastrointestinal anatomy, as between humans and horses.

Several inherent limitations to this study exist because of the nature of working with client-owned animals in a clinical setting. The paired ERU and control horses were housed in similar environmental conditions, but they were not matched based upon signalment or use. These factors have the potential to impact the fecal microbiota beyond expected inter-individual variation and have been previously reported to affect the fecal microbiota in both humans and horses [40,41,42,43,44,45,46,47]. In order to truly exclude the potential impact of these variables, future studies comparing the fecal microbiota between healthy horses and horses with ERU may be warranted with more stringent environment-, diet-, age-, sex-, and breed-matching between groups.

Another study limitation is that there is no standardized treatment for horses with ERU. Client-owned horses may receive a range of medications (e.g., topical and systemic non-steroidal anti-inflammatory drugs (NSAIDs), topical atropine) at differing frequencies based upon clinician preference and disease severity. This variation in medication may also impact the fecal microbiota. For example, systemic administration of NSAIDs causes transient gastrointestinal dysbiosis in horses with decreased alpha diversity and loss of members of the Firmicutes phylum [48]. Additionally, horses were enrolled in the study with fecal samples collected in varying states of disease (i.e., active uveitis versus quiescent periods). This may represent a confounding variable, as the fecal microbiota could potentially vary based upon disease state. This could be addressed in future studies with a larger study sample size through further subdividing the ERU group into active uveitis versus quiescent subgroups during statistical analysis. Alternatively, the fecal microbiota could be assessed in several individuals at multiple time-points (to include uveitic flare-up, recovery, and quiescent periods).

## 5. Conclusions

In conclusion, based upon the results of the present study, there does not appear to be an association between fecal microbiota and ERU. No significant alterations in the fecal bacterial microbiota were noted between horses with equine recurrent uveitis and healthy, environmentally matched controls. Future studies are warranted to assess the fecal microbiota in subgroups of patients with active uveitic flare-ups and quiescent disease. Additionally, metabolomics studies should be performed to characterize the fecal metabolic phenotype of horses with ERU and compare it with healthy controls.

## Figures and Tables

**Table 1:** Description of equine recurrent uveitis (ERU) patient signalment, affected eye(s), intraocular pressure, aqueous flare grade, ophthalmic examination findings, and treatment.

ERU Patient #	Age (Years)	Breed	Sex	Affected Eye(s)	IOP	Aqueous Flare	Ophthalmic Examination Findings	Topical Treatment	Systemic Treatment
1	22	Appaloosa	Gelding	OU	4 mm Hg OD, TSTM OS	3+ flare OU	OD: Mild blepharospasm, epiphora, corneal edema with keratic precipitates, vitreal prolapse, hypermature cataract, posteriorly luxated lens; OS: Absent menace response, dazzle reflex, direct and consensual PLRs, moderate blepharospasm, epiphora, corneal edema with subepithelial mineralization, keratic precipitates, superficial corneal ulcer with infiltrate, vitreal prolapse, retinal degeneration	Atropine 1% o/s OU q12h, diclofenac 0.1% o/s OU q12h, SOCS1 OU q12h, serum OS q4h, cefazolin 5.5% o/s OS q4h, tobramycin 0.3% o/s OS q4h, EDTA 1% o/s OS q6h, SSD 1% OS q6h	Pergolide 0.5 mg PO q24h, firocoxib 28.5 mg PO q24h, Gastrogard (omeprazole) 250 lbs dose PO q24h
2	16	Miniature	Gelding	OU	8 mm Hg OD, 6 mm Hg OS	2+ flare OD, 3+ flare and cell OS	OD: Mild blepharospasm, mild epiphora, phthisis bulbi, episcleral injection, absent menace response, dazzle reflex, direct and consensual PLRs, mild diffuse corneal edema, fibrovascular membrane with extensive posterior synechia, miosis, hypermature cataract; OS: Moderate blepharospasm and epiphora, episcleral injection, absent menace response, direct and consensual PLRs, corneal edema with keratic precipitates, corneal endothelial stria with adherent fibrin, hyphema, miosis	SOCS1 OU q12h, atropine 1% o/o OU q12h	Flunixin meglumine 150 mg PO q24h
3	10	Appaloosa	Gelding	OU	7 mm Hg OU	1+ flare OD, 3+ flare OS	OD: Phthisis bulbi, mild episcleral injection, absent menace response, dazzle reflex, direct and consensual PLRs, corneal edema and fibrosis, posterior synechia, miosis, hypermature cataract; OS: Mild episcleral injection, absent menace response, direct and consensual PLRs, corneal edema, corpora nigra atrophy, miosis	Prednisolone acetate 1% o/s OS q6h, atropine 1% o/o q6h, SOCS1 OU q12h	Flunixin meglumine 250 mg PO q12h
4	18	Appaloosa	Gelding	OU	10 mm Hg OD, 8 mm Hg OS	No flare OU	OD: Blunted corpora nigra, fibrinous material at pupillary margin; OS: Corneal fibrosis, fibrinous material at pupillary margin, incipient cataract	SOCS1 OU q12h	None
5	17	Appaloosa	Mare	OU	13 mm Hg OD, 14 mm Hg OS	Trace flare OD, no flare OS	OD: Mild conjunctival hyperemia, corpora nigra atrophy, incipient cataract, vitreal prolapse in anterior chamber, chorioretinal scars; OS: Corpora nigra atrophy, flocculent debris in anterior chamber, incipient cataract, chorioretinal scars	Flurbiprofen 0.03% o/o OU q12h	None
6	16	Quarter Horse	Gelding	OS	TSTM OS	1+ flare OS	OS: Mild blepharospasm, negative menace response and direct PLR, corneal neovascularization, blunted corpora nigra, miosis, hypermature cataract	Atropine 1% o/o OS q12h, diclofenac 0.1% o/o OS q12h, SOCS1 OS q12h	None
7	23	Thoroughbred	Mare	OU	Not measured OU	No flare OU	OD: Blunted corpora nigra, hyperpigmented iris, vitreal prolapse, immature cataract; OS: Moderate blepharospasm, mild conjunctival hyperemia and episcleral injection, corneal erosion, blunted corpora nigra, hyperpigmented iris, immature cataracts	Serum OS q4–6h, atropine 1% o/s OS q12h	Flunixin meglumine 450 mg PO q12h
8	22	Appaloosa	Mare	OU	TSTM OU	1+ flare OD, No flare OS	OD: Hyperpigmented iris, posterior synechiae, blunted corpora nigra, incipient cataract; OS: Blunted corpora nigra, hyperpigmented iris, incipient cataract and pigment on anterior lens capsule, vitreal degeneration	Atropine 1% o/s OU q12h, neomycin-polymyxin b-dexamethasone 0.1% o/o OU q8h, diclofenac 0.1% o/o OU q8h	Flunixin meglumine 250 mg PO q12h
9	7	Quarter Horse	Mare	OD	12 mm Hg OD	No flare OD	OD: Blunted corpora nigra, hyperpigmented iris, multifocal peripapillary depigmented foci	Neomycin-polymyxin b-dexamethasone 0.1% o/o OD q12h, diclofenac 0.1% OD q12h	None
10	16	Paint	Mare	OD	15 mm Hg OD	1+ flare OD	OD: Mild conjunctival hyperemia and episcleral injection, corneal edema, keratic precipitates, blunted corpora nigra, miosis, immature cataract	Atropine 1% o/s OD q24h, SOCS1 OD q12h	None
11	6	Dutch Warmblood	Gelding	OD	Not measured OD	No flare OD	OD: Keratic precipitates, blunted corpora nigra, hyperpigmented iris, posterior synechia, incipient cataract, multifocal peripapillary depigmented foci	Neomycin-polymyxin b-dexamethasone 0.1% o/o OD q8h, atropine 1% o/o OD q8h	Flunixin meglumine 500 mg PO q12h
12	16	Quarter Horse	Stallion	OU	19 mm Hg OD, 11 mm Hg OS	No flare OU	OD: Inconsistent menace response, absent direct and consensual PLR, corneal edema with striae, blunted corpora nigra, vitreous and fibrin around pupillary margin; OS: Keratic precipitates, blunted corpora nigra, iris hyperpigmentation, posterior synechia, pigment on anterior lens capsule, peripapillary depigmented foci	Diclofenac 0.1% o/o OU q12h, sodium chloride 5% o/o OD q12h	Phenylbutazone 1 mg PO q24h
13	19	Quarter Horse	Gelding	OU	20 mm Hg OD, not measured OS	Trace flare OD, 2+ flare OS	OD: Buphthalmic, episcleral injection, corneal edema, pigment and fibrin on anterior lens capsule, immature cataract; OS: Phthisis bulbi, absent menace response, direct and consensual PLRs, episcleral injection, corneal edema, posterior lens luxation, hypermature cataract	Prednisolone acetate 1% o/o OD q8h, atropine 1% o/o OD q8h, sodium chloride 5% o/o OD q8h	Phenylbutazone 1 mg PO q12h
14	15	Appaloosa	Mare	OU	7 mm Hg OD, TSTM OS	1+ flare OD, 3+ flare OS	OD: Conjunctival hyperemia, absent consensual PLR, keratic precipitates, hyperpigmented iris, inflammatory debris and pigment on anterior lens capsule, incipient cataract, green hue to vitreous; OS: Phthisis bulbi, conjunctival hyperemia, absent direct PLR, corneal edema and neovascularization, inflammatory debris and pigment on anterior lens capsule, lens subluxation, green hue to vitreous	SOCS1 OU q12h, atropine 1% o/o OU q12h, diclofenac 0.1% OU q12h, neomycin-polymyxin b-dexamethasone 0.1% o/o OU q8h	Flunixin meglumine 500 mg PO q12h
15	28	Draft mix	Mare	OS	14 mm Hg OS	Trace flare OS	OS: Negative menace response, direct PLR, conjunctival hyperemia, keratic precipitates, corneal edema, peripupillary fibrosis, rubeosis iridis, blunted corpora nigra, miosis, mature cataract	Diclofenac 0.1% o/s OS q8h	None

Abbreviations: IOP, intraocular pressure; OD, right eye; OS, left eye; OU, both eyes; PLR, pupillary light reflex; TSTM, too soft to measure; SOCS1, suppressor of cytokine signaling 1; o/s, ophthalmic solution; o/o, ophthalmic ointment; PO, *per os*.

## Data Availability

Publicly available datasets were analyzed in this study. This data can be found here: NCBI database, bioproject number 9206854.
